# Optimized somatic embryogenesis and plant regeneration in elite Argentinian sugarcane (*Saccharum* spp.) cultivars

**DOI:** 10.1186/s43141-021-00270-8

**Published:** 2021-11-08

**Authors:** Valentina Di Pauli, Paola Daniela Fontana, Dalia Marcela Lewi, Arturo Felipe, Luis Ernesto Erazzú

**Affiliations:** 1 National Institute of Agricultural Technology (INTA), Experimental Station Famaillá, Tucumán, Argentina; 2 National Institute of Agricultural Technology (INTA), Genetic Institute Ewald A. Favret (IGEAF), Buenos Aires, Argentina

**Keywords:** Biotechnological breeding, Tissue culture, Callus, Somatic embryos, Genotype dependence

## Abstract

**Background:**

Biotechnological breeding of elite sugarcane cultivars is currently limited because of the difficulty of regenerating plants by tissue culture. Here, we report that commercially elite sugarcane genotypes, which are adapted to Argentinian agro-ecological conditions, are capable of being regenerated via indirect somatic embryogenesis. Leaf rolls of five elite genotypes were cultured following two callus induction protocols using different concentrations of 2,4-D as the growth regulator. Embryogenic calluses were regenerated under light conditions. Regenerated plants were subsequently acclimatized in the greenhouse under two acclimatization procedures before being transplanted to the field.

**Results:**

Four of the five genotypes were able to form somatic embryos following the two induction protocols. The variables related to embryogenic callus production were influenced by the interaction between genotype and culture conditions. For plant regeneration, the embryogenic calluses were further cultured on an IBA-supplemented medium, where we observed a high genotype dependence. Calluses from the four cultivars regenerated a good number of plants. With the procedures described here, we obtained more than 90% of well-acclimatized plants both in the greenhouse and in the field.

**Conclusions:**

This protocol provides a simple way to regenerate sugarcane plants through indirect somatic embryogenesis. Also, the results confirm that tissue culture ability is highly genotype-dependent in sugarcane. Our findings suggest that these elite cultivars could be good candidates for biotechnological breeding.

## Background

Sugarcane (*Saccharum* spp.) is an important agroindustrial crop in the tropics and subtropics due to its high sucrose content and increasing interest in its bioenergy potential [1]. Sugarcane accounts for 80% of global sugar and 40% of bioethanol production [2]. Argentina is one of the top ten producing countries [3]. The sugarcane production area covers about 370,000 hectares being concentrated in the Northwest region [4]. Tucumán is the main sugarcane production province in Argentina with 260,800 hectares cultivated in 2020 [5], yielding 1.4 million tonnes of sugar (78% of the total Argentinian production) and more than 300,000 cubic meters of ethanol [6]. The sugarcane agroindustry plays a key role in the province’s economy as well as being an emblem of cultural identity. The National Institute of Agricultural Technology (INTA) is a leading research institute in Argentina. In Tucumán, INTA conducts a nationwide sugarcane breeding program researching the development of new cultivars to enhance sugarcane productivity. INTA has recently incorporated biotechnological approaches for use in sugarcane improvements.

Biotechnology became crucial to face the limitations of classical sugarcane breeding [7]. In this context, in vitro culture systems play an important role in breeding programs. Established protocols for in vitro procedures offer a lot of strategies to deal with the narrow genetic diversity, propagation, and storage limitations curtailing sugarcane industries worldwide [8]. An efficient plant regeneration system for elite genotypes is a requirement for successfully induced mutagenesis, plant transgenesis, or to apply modern techniques such as CRISPR-based genome editing. Indirect somatic embryogenesis is one of the various routes by which sugarcane plants can be regenerated in vitro*.* Through this pathway, somatic embryos originate from a single cell reducing the risk of chimeras among regenerated plants [8, 9].

Ho and Vasil [9] were the first to study the morphology of callus formation and the ontogenesis of somatic embryos in sugarcane. Immature leaves and developing inflorescences are preferred target tissues for the rapid production of embryogenic callus [10–13]. Embryos formation from somatic cells is induced in sugarcane explants in response to auxins, mainly 2,4-dichlorophenoxyacetic acid [13, 14]. Different types of calluses have been described for sugarcane cultivars [10]. Only the compact, hard and yellow callus with smooth-surfaced globular structures that turn white in later stages, shows an embryogenic nature. This embryogenic callus was named Type 3 by Taylor et al. [10].

Somatic embryogenesis is the most common pathway in sugarcane culture with well-established protocols [8]. However, these protocols cannot be used for all sugarcane cultivars [15]. The genotype has an important effect on embryogenic ability in many plant species [16], including sugarcane [11, 12, 14, 17–22]. Furthermore, plant regeneration is a specific and genotype-dependent phenomenon and some sugarcane cultivars are recalcitrant [19, 23]. Therefore, it is necessary to establish an efficient in vitro culture system for each genotype.

The current study assessed the embryogenic potential of elite Argentinian genotypes, which are developed by the Sugarcane Breeding Program of INTA, to identify the most suitable genotypes to apply methods for generating new genetic variability.

## Methods

### Plant materials

The experiment was conducted on five high-yielding commercial cultivars developed (FAM 81-820, INTA CP 98-828, INTA NA 89-686, INTA NA 91-209) or selected (L 91-281) by the Sugarcane Breeding Program of INTA in Tucumán, Argentina. The genotype selection criteria were based on their genetic background and their agronomic performance. The cultivar NA 85-1602, which has shown good in vitro culture performance, was included as reference material. Heat-treated source plants were field cultivated and their phytosanitary condition was controlled before the experiment.

### Establishment of cultures

Cane tops were collected from 7-month-old plants in plant cane. The outer leaves and sheaths were removed, then stalks were cut keeping young internodes and meristematic region. After washing them under running tap water, stalks were sterilized by dipping into ethanol 70% (v/v, 1 min) followed by sodium hypochlorite solution 18 g/L (20 min) under aseptic conditions. They were finally rinsed three times in sterile water. Immature leaf rolls were cut into 1-mm-thick discs and placed on MS3 callus induction medium adding 0.4 g/L cefotaxime as a bacteriostatic agent. MS3 medium consisted of 4.3 g/L basal salts and vitamins (100 mg/L myo-inositol, 0.5 mg/L pyridoxine, 0.5 mg/L nicotinic acid, 1 mg/L thiamine and 2 mg/L glycine) (PhytoTech Labs, USA), supplemented with 20 g/L sucrose, 0.5 g/L casein, 3 mg/L 2,4- dichlorophenoxyacetic acid (2,4-D) and 9 g/L agar (Britania Lab, Argentina), pH 5.8 ± 0.1. The explants were cultured in darkness at 28 ± 2°C. The establishment capacity was recorded after 1 week. The data were expressed as percentage of established explants per total number of explants. An established explant was considered to be the one that initiates callus formation. Sixteen culture plates were used as replicates per genotype with each plate containing 14 calluses.

### Induction of embryogenic callus

We assessed two protocols to induce embryogenic calluses: (a) the explants were cultured for 8 weeks on MS3 medium, named MS3 protocol and (b) the explants were cultured for 4 weeks on MS3 followed by 4 weeks on MS1 medium (like MS3 but with 1 mg/L 2,4-D), named MS3/MS1 protocol. The calluses were cultured in darkness at 28 ± 2°C and subcultured fortnightly. The embryogenic capacity was recorded after 8 weeks. The data were expressed as percentage of calluses with embryogenic response per total number of calluses. An embryogenic callus (Type 3) was identified by its white, compact, and nodular appearance. Also, each callus was visually assessed according to the percentage of Type 3 callus in the total callus volume (PT3) using the scoring system: 1 = 0–25%; 2 = 25–50%; 3 = 50–75% and 4 = 75–100% of PT3. The score per experimental unit was calculated as the average value of total calluses on the plate. Eight culture plates were used as replicates per treatment (induction protocol × genotype) with each plate containing 14 calluses.

### Plant regeneration and acclimatization

Embryogenic calluses were transferred to the regeneration medium RM consisting of the same components as MS3 but with 5 g/L agar and without 2,4-D. RM was supplemented with 5 mg/L indole-3-butyric acid (IBA) every 4 weeks. Plant regeneration was induced with artificial light (dual-lamp fluorescent lighting providing 3678 Lux) in a growth chamber (Thermo Scientific Precision 818) with a 16/8 h (light/dark) photoperiod at 28 ± 2°C. Regenerated plants were subcultured fortnightly on fresh medium. The regeneration capacity was recorded after 12 weeks as the number of plantlets per plate.

When plantlets had reached 6-7 cm in height, they were individually planted into a mixture of peat, sterile soil, and perlite (4:2:1 v/v) and placed in the glass greenhouse under natural conditions of temperature, humidity, and photoperiod (15–28°C, 70–80% humidity and 12.6–14.2 h of light). We assessed two procedures for the acclimatization: (a) regenerated plantlets were transferred to transparent pots and were kept during the first week under 97–98% humidity by covering with a thin plastic and (b) plantlets were transferred to plastic seedling trays without covering under greenhouse humidity (70–80%). The acclimatization capacity was recorded after 2 months. The data were expressed as percentage of acclimatized plantlets per total number of regenerated plantlets. The experimental design was completely randomized with eight replicates of 10 plantlets per treatment (acclimatization procedure × induction protocol × genotype).

One thousand 3-month-old plants were transplanted to the field in a completely randomized experimental design (60 cm plant to plant and 160 cm between rows distance) at Experimental Station Famaillá of INTA, in Tucumán. Field survival was recorded after 2 months. The data were expressed as percentage of surviving plantlets per total plantlets transplanted. Five replicates of 20 plantlets per treatment (acclimatization procedure × genotype) were evaluated.

### Statistical analysis

Generalized linear models were used for data analyses [24]. The establishment capacity, the embryogenic capacity, the acclimatization capacity, and the field survival were analyzed using a binomial error distribution with a logit link function. These variables were transformed into frequencies for statistical analyses. The PT3 was analyzed using a normal error distribution with an identity link function and normality was checked by the Shapiro-Wilks test. The regeneration capacity was analyzed using a negative binomial error distribution with a log link function. Overdispersion was checked with the following formula: Ʃ Pearson residuals^2^/degrees of freedom ≈ 1 [25]. The DGC test [26] was used to determine significant differences between levels of each factor. Also, the correlations between the establishment capacity, the embryogenic capacity, the PT3, and the regeneration capacity were evaluated using the Spearman coefficient (*r*). All data were analyzed using Infostat [27] with an interface with the software package R version 3.4.2 (The R-Foundation for Statistical Computing 2018). *P* ≤ 0.05 was considered statistically significant.

## Results

### Callus induction and somatic embryogenesis

The ability of explants to form callus was evaluated on an induction medium with 2,4-D. An established explant was considered to be the one that initiates callus formation after 1 week, which was shown by the swelling of tissues accompanied by slight oxidation. All genotypes showed a high establishment capacity, greater than 90% (Table 1). However, it was significantly lower in cultivars INTA NA 89-686 (92.56%) and FAM 81-820 (94.31%) than the reference material NA 85-1602 (99.13%) and the other genotypes (*P* < 0.05). In the first days, explant browning was observed in most cultivars, being highest in INTA NA 89-686 and NA 85-1602. This browning vanished during callus proliferation without any effect on embryogenic callus formation.

**Table 1:** Establishment capacity in sugarcane cultivars cultured on callus induction medium MS3 with 3 mg/L 2,4-D and 0.4 g/L cefotaxime

Genotype	Establishment capacity (%)
NA 85-1602	99.13 ± 0.60 a
FAM 81-820	94.31 ± 2.04 b
INTA CP 98-828	99.13 ± 0.60 a
INTA NA 89-686	92.56 ±1.75 b
INTA NA 91-209	99.56 ± 0.44 a
L 91-281	98.69 ± 0.71 a

Means followed by the same letter do not differ according to the DGC test (*P *< 0.05)

The five genotypes were able to form some type of callus on a medium supplemented with 2,4-D. Callus formation began around seven days after the explant establishment. Two protocols were assessed to induce embryogenic calluses. These protocols differed in 2,4-D concentrations throughout the assay. Following MS3 protocol, 2,4-D concentration was kept at 3 mg/L for 8 weeks, whereas following MS3/MS1 protocol 2,4-D concentration was lowered to 1 mg/L in the fourth week. Since a lower auxin concentration may be required for embryo maturation. After 8 weeks, three types of callus were identified: a mucilaginous, semi-translucent, and non-embryogenic callus; a friable and yellowish non-embryogenic callus; and a compact, hard, yellow callus with smooth-surfaced globular structures (somatic embryos), known as Type 3 callus in sugarcane [10]. The frequency of each type in the total callus volume was influenced by genotype and culture conditions. Data analysis of the Percentage of Type 3 callus (PT3) revealed a significant interaction (*P* < 0.05) between genotype and callus induction protocol. Only INTA CP 98-828 showed the same performance as the reference material following both MS3 and MS3/MS1 protocols. INTA CP 98-828 showed a high PT3 greater than 75%, whereas INTA NA 89-686 and INTA NA 91-209 showed a PT3 greater than 50% of callus mass. These genotypes performed similarly in both protocols. In contrast, the cultivar L 91-281 showed around 50% of embryogenic callus in MS3/MS1 protocol but showed a lower PT3 in MS3 protocol (Table 2). The remaining portion of callus mass was mostly friable and yellowish. Calluses from FAM 81-820 were mucilaginous and semi-translucent, without somatic embryos.

The capacity to form embryogenic callus was variable, ranging from 0 to 100%. Four of the five INTA genotypes were able to form somatic embryos: INTA CP 98-828, INTA NA 89-686, INTA NA 91-209, and L 91-281 (Table 2). Like PT3, a significant interaction (*P* < 0.05) was revealed between genotype and protocol regarding embryogenic capacity. INTA CP 98-828 with MS3/MS1 protocol and INTA NA 89-686 with both protocols were the best genotype/protocol combinations, greater than 90% (Table 2). INTA CP 98-828 in MS3 protocol and INTA NA 91-209 in both protocols showed an embryogenic capacity greater than 75%, followed by L 91-281 showing an intermediate capacity in both protocols. FAM 81-820 was unable to form some embryogenic callus and did not regenerate any plants later. Although genotypes differed in their embryogenic capacity following MS3/MS1 protocol, a better somatic embryos maturation was observed in all genotypes under magnification (×40).

**Table 2:** Embryogenic capacity, percentage of Type 3 callus, and regeneration capacity in sugarcane cultivars cultured following two callus induction protocols: MS3 (3 mg/L 2,4-D for 8 weeks) and MS3/MS1 (3 mg/L 2,4-D for 4 weeks, 1 mg/L 2,4-D for 4 weeks)

Genotype	Protocol	Embryogenic capacity (%)	Percentage of Type 3 callus*	Regeneration capacity (number of plantlets/plate)
NA 85-1602	MS3	96.00 ± 2.08 a	3.96 ± 0.04 a	202.50 ± 15.87 a
FAM 81-820		0.00 ± 0.00	0.00 ± 0.00	00.00 ± 00.00
INTA CP 98-828		84.50 ± 3.97 b	4.00 ± 0.04 a	170.86 ± 9.88 a
INTA NA 89-686		94.83 ± 2.37 a	3.25 ± 0.04 b	119.20 ± 16.27 b
INTA NA 91-209		79.13 ± 2.62 b	3.16 ± 0.04 b	135.50 ± 12.36 b
L 91-281		36.00 ± 5.29 c	1.66 ± 0.04 e	41.88 ± 6.72 c
NA 85-1602	MS3/MS1	90.00 ± 2.90 a	3.96 ± 0.04 a	190.00 ± 12.59 a
FAM 81-820		0.00 ± 0.00	0.00 ± 0.00	00.00 ± 00.00
INTA CP 98-828		96.00 ± 2.06 a	4.00 ± 0.04 a	181.43 ± 12.12 a
INTA NA 89-686		89.75 ± 4.26 a	3.26 ± 0.04 b	130.71 ± 17.31 b
INTA NA 91-209		86.88 ± 2.62 b	3.03 ± 0.04 c	138.43 ± 6.99 b
L 91-281		45.25 ± 2.43 c	1.93 ± 0.04 d	53.00 ± 4.51 c

Means followed by the same letter do not differ according to the DGC test (*P *< 0.05)

*Visual scale 1 = 0–25%; 2 = 25–50%; 3 = 50–75%; 4 = 75–100%

### Plant regeneration and ex vitro adaptation

The somatic embryos germination began on the regeneration medium under light conditions and in the absence of 2,4-D. This medium was supplemented with IBA to promote root development. Well-developed plantlets were obtained after 8–12 weeks. Data analysis revealed highly significant differences between the genotypes (*P* < 0.05) and no significant differences between the induction protocols (*P* > 0.05) and interaction effect (*P* > 0.05) regarding regeneration capacity. The cultivar INTA CP 98-828 showed the best regeneration response, with a mean of 176 plantlets/plate regenerated from embryogenic calluses, followed by INTA NA 91-209 (137 plantlets/plate), INTA NA 89-686 (125 plantlets/plate) and L 91-281 (47 plantlets/plate) (Table 2). Abnormal plants, such as albino or variegated plants, were not observed.

**Fig. 1:**
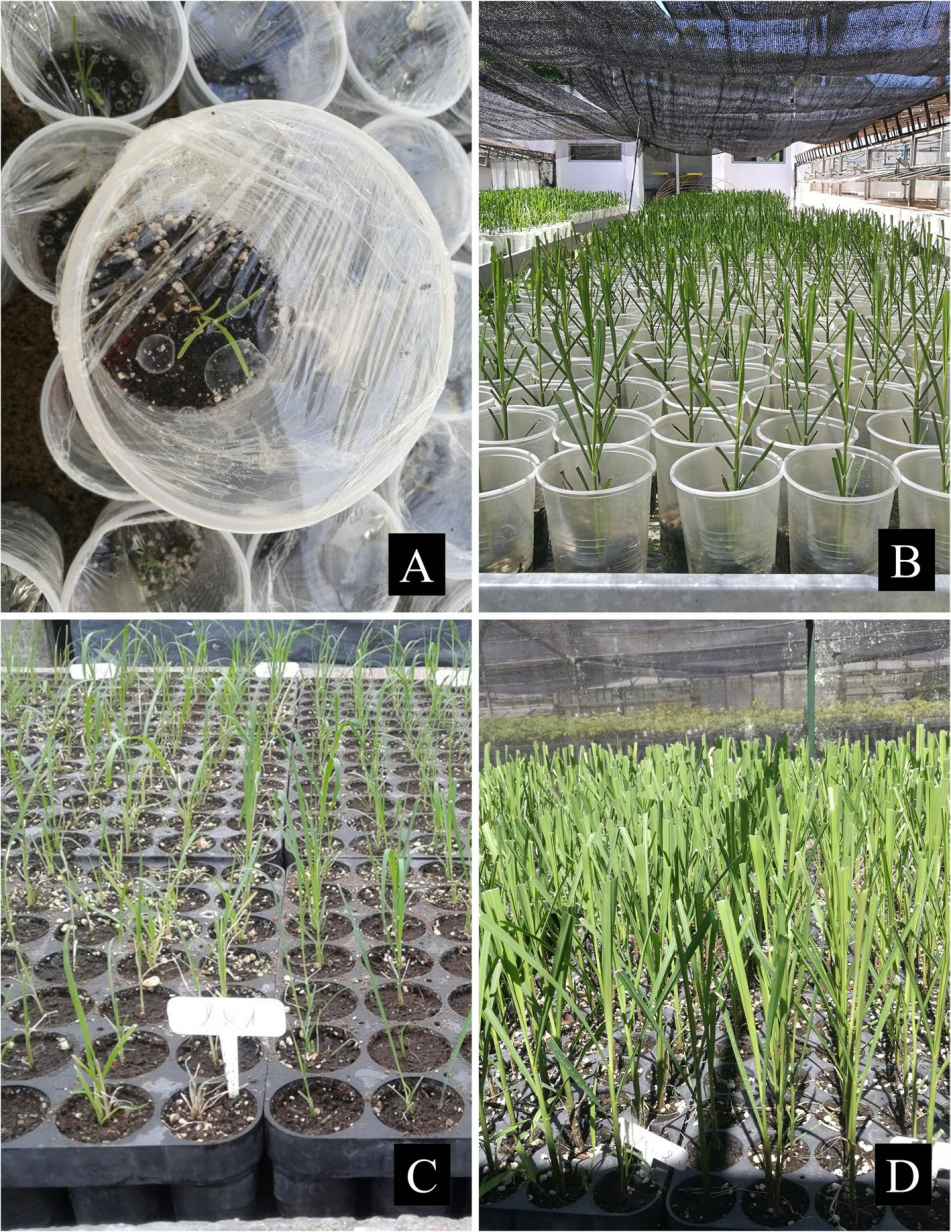
Procedures for the acclimatization. **A**, **B** Plants acclimatized in transparent pots covering with a thin plastic for 1 week. **C**, **D** Plants acclimatized in plastic seedling trays without covering

The regenerated plants were transplanted to the greenhouse for acclimatization. Two acclimatization procedures were assessed. On the one hand, plantlets were acclimatized in transparent pots keeping a high humidity by covering with a thin plastic (Fig. 1A, B). On the other hand, plantlets were acclimatized in plastic seedling trays without covering under greenhouse humidity (Fig. 1C, D). There were no significant differences between the genotypes (*P* > 0.05), but there were slight differences between acclimatization procedures (*P* < 0.05). Although acclimatization was slightly better on trays (96.80%) than on pots (93.91%), all genotypes were successfully adapted in ex vitro conditions with an acclimatization capacity greater than 90% (Table 3). Three-month-old plants were transferred to the field after initial acclimatization. All genotypes were successfully acclimatized to field conditions with a survival greater than 98% (Table 3).

**Table 3:** Acclimatization capacity and field survival in sugarcane cultivars cultured acclimatized in pots with covering or trays without covering

Genotype	Protocol	Acclimatization capacity (%)	Field survival (%)
NA 85-1602	Pot	95.73 ± 1.07 a	100.00 ± 0.00 a
INTA CP 98-828		93.43 ± 1.74 a	99.00 ± 1.00 a
INTA NA 89-686		94.25 ± 1.89 a	97.00 ± 2.00 a
INTA NA 91-209		95.23 ± 1.62 a	99.00 ± 1.00 a
L 91-281		91.13 ± 2.48 a	99.00 ± 1.00 a
NA 85-1602	Tray	96.47 ± 1.34 b	98.00 ± 2.00 a
INTA CP 98-828		96.86 ± 1.38 b	99.00 ± 1.00 a
INTA NA 89-686		96.67 ± 1.42 b	100.00 ± 0.00 a
INTA NA 91-209		95.38 ± 2.20 b	100.00 ± 0.00 a
L 91-281		98.40 ± 1.09 b	100.00 ± 0.00 a

Means followed by the same letter do not differ according to the DGC test (*P *< 0.05)

### Correlation analyses

A positive correlation was found between the regeneration capacity and the percentage of embryogenic callus (*r* = 0.77; *P* < 0.05), between the regeneration capacity and the embryogenic capacity (*r* = 0.79; *P* < 0.05) as well as between the percentage of embryogenic callus and the embryogenic capacity (*r* = 0.59; *P* < 0.05). No significant correlation was found between the establishment capacity and the other variables (*P >* 0.05).

## Discussion

An efficient plant regeneration system for elite genotypes is essential to apply biotechnological breeding methods. Somatic embryogenesis is a rapid and non-chimeric pathway for plant regeneration in sugarcane in this respect. However, it is limited by low regeneration efficiency and strong genotype-dependent effects. Even though many established protocols are available for sugarcane, not all are adapted to elite cultivars. Usually, biotechnological breeding procedures involve the use of genotypes that have good characteristics for in vitro culture but poor adaptation to Argentinian agro-ecological conditions. In the current study, we identified the in vitro culture response of five commercially grown Argentinian sugarcane cultivars through indirect somatic embryogenesis. These cultivars were developed by the Sugarcane Breeding Program of INTA and are considered elite genotypes. We based on Snyman [28] protocol for genetic transformation with some modifications according to other studies and our conditions.

A range of explants has been tried in somatic embryogenesis studies in sugarcane [11, 13]. Immature leaf roll and developing inflorescence are the best explants for embryogenic callus production. We used immature leaf explants and achieved a successful in vitro establishment in our genotypes. In Tucumán, where sugarcane flowering does not occur naturally, it is easier to work with leaf roll than immature inflorescence as explant, thereby we can dispense with inducing flowering. Non-flowering clones can also be found among commercially elite genotypes [29]. Furthermore, explant contamination was almost absent following a disinfection protocol, such as that proposed by Ho and Vasil [9].

Embryogenic calluses are the best target tissue for in vitro mutagenesis [30] and biolistic transformation [12, 28] as well as cryoconservation and synthetic seed production [8] in sugarcane. Therefore, the nature and type of callus are some of the most important factors to consider in sugarcane tissue culture. Explants of all genotypes formed the different types of calluses described by Taylor et al. [10] with the two protocols we assessed. A highly embryogenic callus was observed in four INTA genotypes, which showed a differential response to induction protocols. Our results are in agreement with other studies demonstrating that embryogenic capacity depends on the genotype and culture medium interaction [13, 31]. Genotype dependence for embryo formation has been reported for other elite sugarcane cultivars in Brazil [14, 15, 32], Venezuela [17], South Africa [12], India [19, 20], Pakistan [21], and the USA [18]. According to the results of the present study, the cultivars INTA CP 98-828, INTA NA 89-686, and INTA NA 91-209 could be classified as highly embryogenic, L 91-281 as moderate, and FAM 81-820 as recalcitrant cultivar with MS3/MS1 and M3 protocols. It is worth noting that other medium formulations, mainly with other growth regulators, could increase embryogenic capacity in L91-281 and induce somatic embryogenesis in FAM 81-281. Cytokines such as kinetin or 6-benzylaminopurine increased embryogenic response in some sugarcane genotypes [13, 19], whereas they decreased it in others [14]. Some cultivars showed a better response to picloram or dicamba than to 2,4-D [17, 21]. A recent study has shown that low concentrations of methylglyoxal enhanced somatic embryogenesis in Indian sugarcane cultivars [33].

A better embryos maturation was observed in MS3/MS1 protocol than MS3 protocol, most likely because of the lower concentration of 2,4-D during the last weeks of culture. The dedifferentiation process and the embryogenic-cell initiation require relatively high auxin concentration, whereas lower auxin concentration may be required for advanced embryogenesis stages [16]. Well-developed embryos are obtained from embryogenic callus when 2,4-D in the medium is lowered [9]. Dibax et al. [32] observed that a decrease in 2,4-D concentration doubled the number of embryogenic masses in Brazilian genotypes. However, we did not observe an increase in the percentage of embryogenic callus with MS3/MS1 protocol.

Light is a major factor affecting somatic embryogenesis and subsequent plant regeneration [34]. Darkness is required for the somatic embryo initiation to keep high auxin concentrations since the auxins are generally prone to breakdown in light [35]. In contrast, light conditions and low auxin levels are necessary for embryo maturation and when the plant becomes autotrophic. Also, the type of light affects these stages, since specific light spectral ranges are involved in specific plant responses. In sugarcane, fluorescent lamps were more efficient in embryo maturation and regeneration, whereas LED-light emitting diodes provided higher multiplication rates [34]. In the current study, we used only fluorescent lamps, but we will subsequently evaluate the multiplication rate of these cultivars using LED lights.

Several factors are considered for the selection of a genotype for biotechnological breeding, mainly its ability to regenerate plants. The germination of somatic embryos in sugarcane can be induced under light conditions and without auxins [9, 10]. We added IBA to promote root development in agreement with Mustafa and Khan [36], whereas other studies managed to regenerate plants without growth regulators [12, 17, 18, 32] or with some cytokine [13, 14]. The ability to form regenerable calluses is genotype-dependent in many species [16], including sugarcane [19, 37]. Our results, which are in agreement with these previous studies, confirm the genotype-dependent regeneration in sugarcane. Although the germination percentage of somatic embryos is very low in some crops [30], we obtained a good number of plants per genotype. In vitro regenerated plants were successfully acclimatized in the greenhouse and later under field conditions. Acclimatization in trays advantaged the stem girth increasing and avoided fungus proliferation when compared with pot culture conditions under high humidity. We found a positive correlation between variables related to embryogenic callus production and plant regeneration, thus suggesting that the regeneration potential of a cultivar can be assessed through the type of callus it produced. No correlation was found between regeneration capacity and establishment capacity because these pathways are controlled for different mechanisms [18].

## Conclusions

The current study shows a highly efficient regeneration system via somatic embryogenesis and the protocols described here can be used to optimize in vitro culture in other sugarcane cultivars. Furthermore, this is the first report of somatic embryogenesis response in Argentinian sugarcane cultivars developed by INTA. These results will allow us to apply biotechnological approaches in our sugarcane breeding program.

## Data Availability

The datasets generated and analyzed during the current study are available from the corresponding author on reasonable request.

## Abbreviations

2,4-D: 2,4-dichlorophenoxyacetic acid
DGC test: Di Rienzo, Guzmán, and Casanoves test
IBA: Indole-3-butyric acid
INTA: National Institute of Agricultural Technology
RM: Regeneration medium
MS3: Callus induction medium with 3 mg/L 2,4-D
MS1: Callus induction medium with 1 mg/L 2,4-D
PT3: Percentage of Type 3 callus in the total callus volume
